# The relationship between safety measures, preparedness, and mental health outcomes in New York City during the COVID-19 pandemic

**DOI:** 10.3389/fpsyt.2025.1547178

**Published:** 2025-04-09

**Authors:** Thi Kim Ngan Vo, Norbert Skokauskas, Keely Cheslack-Postava, Christina W. Hoven

**Affiliations:** ^1^ Department of Public Health and Nursing, Norwegian University of Science and Technology (NTNU), Trondheim, Norway; ^2^ Regional Centre for Child and Youth Mental Health and Child Welfare, Department of Mental Health (IPH), NTNU, Trondheim, Norway; ^3^ Global Psychiatric Epidemiology Group, Division of Child and Adolescent Psychiatry, Department of Psychiatry, Columbia University-New York State Psychiatric Institute, New York, NY, United States; ^4^ Department of Epidemiology, Columbia University Mailman School of Public Health, New York, NY, United States

**Keywords:** anxiety, COVID-19, depression, New York City, pandemic responses, preparedness, safety measures, time trends

## Abstract

**Background:**

The COVID-19 pandemic necessitated strict safety measures and preparedness, potentially influencing mental well-being worldwide. This study investigated the impact of safety measures and preparedness levels on mental health outcomes during the COVID-19 pandemic in New York City, USA examining how sociodemographic characteristics modified these associations.

**Method:**

A longitudinal study of 1,227 participants from three ongoing cohorts, provided data through telephone interviews across three waves from March 2020 to August 2021. Depression and anxiety were measured using Patient Health Questionnaire 8 (PHQ-8) and Generalized Anxiety Disorder 7 (GAD-7). Logistic regression models were used to investigate associations between safety measures, preparedness, and mental health outcomes over time, adjusting for potential confounders and assessing the modification effect of demographic factors.

**Results:**

At Wave 1, 18% of participants reported moderate to severe depression, while 20% had moderate to severe anxiety. Over time, these rates declined significantly, with depression dropping to 9% and anxiety to 10% by Wave 3. Safety measures practiced at Wave 1 showed a protective effect on depression at Wave 3 (OR=0.45, 95% CI: 0.22, 0.91). Higher preparedness levels were significantly associated with reduced odds of anxiety (aOR=0.72, 95% CI: 0.55, 0.93) in the concurrent wave. Age specific analysis revealed that individuals aged 26-35 experienced stronger protective effects from higher preparedness levels (OR=0.43, 95% CI: 0.2, 0.92) compared to younger age groups.

**Conclusion:**

This study highlights the importance of safety measures and preparedness in mitigating mental health challenges during crises. Addressing age specific factors and preparedness levels can guide the public health strategies to better support diverse populations.

## Introduction

The COVID-19 pandemic triggered a significant rise in mental disorders, particularly depression and anxiety, attributed to multiple stressors including fear of infection, economic instability, and social isolation (1). These disorders reflected a complex interplay of environmental, social, and biological factors (2). Longitudinal evidence revealed heterogeneous mental health trajectories across populations, with New York City showing declining rates of moderate to severe depression from March 2020 to February 2021 (3, 4), while other regions experienced fluctuating patterns of mental health outcomes and sustained increased in mental health service utilization (5, 6). In response to the pandemic, safety measures were introduced to limit the spread of COVID-19 transmission (7). Although preventive behaviors such as avoiding face-touching (8) and maintaining hand hygiene (9) were widely adopted, their psychological implications manifested differently. For example, after the measures were introduced Ghassemi et al. reported that adherence to increased handwashing, positively correlated with anxiety but demonstrated varied effects on depression (10). The efficacy and psychological impact of practicing safety measures also differed among individuals with pre-existing mental health disorders and the general population (11). Research revealed that individuals with anxiety exhibited heightened adherence to hygiene practices, however they paradoxically engaged in more conscious face touching, potentially elevating the risk of infection (11). The adoption of safety measures was modulated by various psychological factors. Evidence indicates that fear of COVID-19 and anxiety about the disease functioned as a predictor of safety behaviors implementation (12, 13). This association suggests that safety measures provided individuals with a sense of control and psychological reassurance during the pandemic. While hand hygiene and the use of personal protective equipment emerged as essential tools for COVID-19 prevention (14), the cognitive demands associated with strict adherence potentially outweighed their perceived benefits (15, 16). Preparedness defined as the readiness and capacity of individuals, households, and communities to respond effectively to the pandemic (17, 18), emerged as factors influencing mental health outcomes. Preparedness is multidimensional, involving access to essential supplies, resources, and contingency plans (19, 20). While perceptions of preparedness can influence psychological well-being, with low perceived preparedness linked to higher stress and burnout, particularly in healthcare settings (21), evidence suggests that traditional preparedness measures alone may not guarantee improvements in mental health outcomes (22). For instance, standard preparedness measures, such as emergency kits, evacuation plans and supplies medication did not consistently prevent adverse outcomes, including increased stress, anxiety and utilization of mental health services (22).

Socioeconomic factors emerged as key determinants in shaping mental health outcomes (23), influencing an individual’s ability to adapt during crisis. A study examined COVID-19 related concerns in New York City found that lower income group report significantly high levels of concern about economic issues such as paying bills, unemployment, and food security compared to higher income groups (24). These disparities disproportionately affected minority and low-income communities, with Black and Hispanic populations experiencing high levels of financial stress and food insecurity (25). A longitudinal study in New York City metropolitan area revealed that while anxiety levels generally decreased over time for most of racial groups, they remained persistently elevated among Asian participants throughout the first year of the pandemic (26). Additionally, younger populations, especially adolescents and young adults, faced heightened vulnerability to anxiety and depression due to disruptions in education, social isolation, and employment uncertainty (27).

This study aims to investigate the relationships between safety measures, preparedness, and mental health outcomes in a heterogeneous population significantly affected by the pandemic, with a particular focus on how demographic factors such as age, race, and income modify these relationships. By employing a longitudinal approach, this study seeks to provide an understanding of changes in the associations of safety and preparedness measures with mental health outcomes through different pandemic phases, ultimately informing public health strategies that balance disease control with mental health and address the needs of diverse populations.

## Material and methods

### Participants

As described previously (3, 4, 24, 26), this longitudinal study used data from a survey including participants randomly selected from three ongoing cohorts based in the New York City metropolitan area. Two of the cohorts included individuals directly or indirectly exposed to the 9/11 attacks, including those with parental exposure. The third cohort focused on individuals from the South Bronx, specifically individuals with parental involvement in the criminal justice system. Each cohort enrolled demographically and geographically matched individuals without 9/11 or criminal justice system exposure were included, along with parents of primary participants (26).

Data were collected through telephone surveys over three waves at six-month intervals during the COVID-19 pandemic: Wave 1 (March-August 2020), Wave 2 (September 2020 - February 2021), and Wave 3 (March-August 2021). Interviews were conducted by trained telephone interviewers who were blinded to participants’ original cohort assignments to ensure unbiased data collection. Participants selected their preferred language for the interview, with most conducted in English (91%), followed by Spanish (8%) and Mandarin (1%). Responses were entered directly into secure platform: Quartics for baseline data and REDCap for follow up waves. The questionnaire was developed to assess mental health and experiences related to COVID-19.

Ethical approval for the original study was granted by the Institutional Review Board of the New York State Psychiatric Institute (protocol #8051), with expedited approval for the baseline and full approval for subsequent waves. Informed consent was obtained via telephone for baseline interviews, as all participants had previously consented to be contacted for future research. Written consent was obtained for follow-up interviews via REDCap. The study adhered to the ethical standards outlined in the Declaration of Helsinki (1975, revised 2008). The current report is based on secondary analysis, defined as the reuse of existing cohort data to address new research questions related to COVID-19. Ethical approval for this secondary analysis was also granted by the Regional Committee for Medical Research Ethics (REK) in Norway on September 14, 2023 (Approval Number: 600769).

A total of 1,227 participants were initially included in the study, five were excluded due to missing data in Wave 1. By Wave 2, 927 participants remained, and 815 completed Wave 3, reflecting dropout rates of 24% and 12%, respectively.

### Measurements

*Mental health outcomes* were measured repeatedly across three waves.

Depressive symptoms were assessed using the Patient Health Questionnaire (PHQ-8) a validated instrument for assessing depression severity (28). The PHQ-8 consists of eight items, each item is rated on a scale from 0 (not at all) to 3 (nearly every day), reflecting the participant’s experiences over the previous two weeks. The total score, ranging from 0 to 24, is calculated by summing the individual item scores, with higher scores indicating greater depression severity. A cut-off score of 10 or higher was used to identify moderate to severe depression (28).

Anxiety symptoms were evaluated using the Generalized Anxiety Disorder-7 (GAD-7) scale. The GAD-7 comprises seven items, each scored on a four-point scale from 0 (not at all) to 3 (nearly every day), reflecting the frequency of anxiety symptoms (29). The total GAD-7 scores range from 0 to 21, with participants scoring 10 or higher classified as experiencing moderate to severe anxiety (29).

*Safety Measures* were assessed using three binary questions collected during Wave 1. Participants were asked whether they had: reduced the number of times they touched their face, increased the frequency of daily handwashing, and used hand sanitizer daily, since the onset of the pandemic. The level of adherence was quantified by summing the affirmative responses, resulting in scores ranging from 0 to 3, with higher scores reflecting greater adherence.

*Preparedness level* was assessed as a time-varying exposure across Waves 1, 2, and 3 using six dichotomous questions evaluating different dimensions of participants’ readiness. These included having access to a private room for quarantine, sufficient food and water, non-perishable canned food, prescription medication, medical equipment, and childcare supplies. Participants responded “Yes”, “No” or “Not applicable” to each items. Preparedness scores were calculated by summing items where participants responded “Yes” or “Not applicable”, resulting in a preparedness score ranging from 0 (completely unprepared) to a maximum score of 5 (fully prepared). Higher scores indicated greater level of preparedness.

*Covariates* were collected at Wave 1. These characteristics included age, gender, race and ethnicity, household income, marital status, and medical conditions, all of which were self-reported by participants. The original cohort assignment and respondent types (youth or parent) were included as covariates in the analysis.

### Statistical analysis

Preliminary analyses summarized the prevalence of moderate to severe depression, anxiety, adherence to safety protocols, and preparedness levels across all waves.

Bivariate tests (t-tests, ANOVA tests and chi-square tests) were conducted to evaluate associations between demographic variables and both exposures (safety measures, preparedness scores) and outcomes (depression, anxiety) at Wave 1. The identification of potential confounders for further analysis was based on the combination of statistical and theoretical considerations.

For the relationship between safety measures and mental health outcomes, gender and type of cohorts were selected as potential confounders. Gender was marginally associated with safety measures (p=0.06), and significantly associated with anxiety (p=0.04). Type of cohorts was retained due to its significant association with safety measures (p=0.02) and its representation of study populations with various trauma exposures. Previous research has established that past trauma can significantly influence mental health outcomes during subsequent crises, such as COVID-19 (30).

For the relationship between preparedness and mental health outcomes, household income and marital status were chosen based on their significant association (p<0.05) with both exposure (preparedness) and at least one of the outcomes.

Missing values for bivariate tests were handled using available-case analysis approach, where observations excluded only from analyses requiring the missing variables. This method allowed for a practical balance between maximizing participant retention and minimizing bias due to the missingness.

Mixed effects logistic regression models were used to assess individual time trends in mental health outcomes and their association with safety measures and preparedness. This approach was chosen because it accounts for to the hierarchical structure of this data, with repeated observations nested within individual over three waves (26). They are particularly well suited for longitudinal data as they allow for inclusion of participants with incomplete observations at some waves (i.e., handling missing values through available case analysis) while preserving statistical power (31). Additionally, these models offer the flexibility in handling unbalanced data resulting from attrition and allow for the inclusion of time varying exposure (31). In these models, random intercepts accounted for individual baseline differences and wave treated as a continuous variable to capture linear trends in mental health outcomes across all waves. The analysis focused on adjusted models, which incorporated relevant covariates and exposure-time interaction terms. These interaction terms were computed by multiplying the safety measure or preparedness scores by the wave, allowing for the examination of the potential moderating effect of time on the associations between safety measures and preparedness with mental health outcomes. A significance threshold of p<0.05 was used to evaluate the interaction terms. Only interaction terms meeting this significance level were retained in the final model.

To explore potential heterogeneity in how age, race/ethnicity, and income moderated these relationships, a series of mixed effects logistic regression models were fitted. Specifically, separate models were estimated for each exposure-outcome combination, including relevant covariates and interaction terms between the exposures and each demographic factor (age, race/ethnicity and income). Non-significant interactions (p>0.05) were noted but not presented in the final results.

All statistical analyses were conducted using R version 4.3.2 (32).

## Results

### Participant characteristics, prevalence of exposures and mental health outcomes

The final sample included 1,222 participants (**Table 1**), with 41% (n=449) from the cohort based on direct exposure to the 9/11 attacks during childhood (The Stress and Wellbeing cohort-SW), 38% (n=465) from the cohort based on parental exposure to 9/11 (The First Responders cohort-FR), and 21% (n=258) from the cohort based on parental exposure to the criminal justice system (The Stress and Justice cohort-SJ). The sample was predominantly female (n=771, 63%). Parents made up 56% of the sample (n=689, mean age=56.4 years), whereas young adults accounted for 44% (n=533, mean age=25.1 years).

**Table 1:** Characteristics of the study participants.

Characteristic	Frequency	Relative Frequency (%)
Types of Cohorts		
SW	499	41%
FR	465	38%
SJ	258	21%
Genders		
Male	451	37%
Female	771	63%
Respondent types		
Parents (mean age=56,4)	689	56%
Young adults (mean age=25,1)	533	44%
Race and ethnicity^a^		
Asian not Hispanic	73	6%
Black/African American, not Hispanic	87	7%
Hispanic	351	29%
White	555	46%
Other/Unknown	141	12%
Age		
15-25	321	26%
26-35	200	16%
36-50	167	14%
51-65	452	37%
66 and above	82	7%
Household income^b^		
Low (<$35K)	175	16%
Middle ($35-100K)	367	33%
High (>$100K)	556	51%
Marital status^c^		
Married or living with partner	577	47%
Never married	422	35%
Divorced; Widowed; Separate	147	12%
Other	75	6%
Chronic medical conditions^d^		
Yes	288	28%
No	759	72%

a Missing values for race and ethnicity: 15.

b Missing values for household income: 124.

c Missing values for marital status: 1.

d Missing values for medical condition: 175.

The racial and ethnic composition of the participants was diverse: white individuals constituted the majority (n=555, 46%); followed by Hispanic participants (n=351, 29%); meanwhile, Black or African American and Asian participants made up 7% (n=87) and 6% (n=73) of the sample, respectively; the remaining 12% (n=141) were classified as belonging to other groups.

Most participants were aged 51-65 years (37%, n=452), followed by those aged 15-25 years (26%, n=321). The 26-35 and 36-50 age groups comprised 16% (n=200) and 14% (n=167), respectively, while those aged 66 and above represented 7% (n=82).

Socioeconomic status was assessed by household income: high-income (> $100,000) made up 51% of the sample (n=556), middle-income ($35,000 - $100,000) accounted for 33% (n=367), and low-income (< $35,000) constituted 16% (n=175). Regarding marital status, nearly half were married or living with a partner (47%, n=577), while 35% had never been married. The remainder were divorced, widowed, separated, or reported other statuses.

At Wave 1, adherence to safety measures was distributed with 52% of participants (n=633) adhering to all three measures. The distribution of preparedness score (**Table 2**) changed over the three waves, with the proportion of fully prepared participants (score 5) increasing from Wave 1 (49%) to Wave 2 (52%) and Wave 3 (56%) while those with minimal preparedness (score 0-1) remained consistently low across all waves. The mean preparedness scores also increased slightly, from 4.22 (SD=0.96) in Wave 1 to 4.32 (SD=0.87) in Wave 2, and 4.36 (SD=0.87) in Wave 3, reflecting improved preparedness levels over time. The prevalence of depression and anxiety decreased over time, with depression dropping from 18% to 9% and anxiety from 20% to 10% across three waves.

**Table 2:** Distribution of safety measures, preparedness scores and mental health outcomes.

	*Wave 1 (N=1222)*	*Wave 2 (N= 927)*	*Wave 3 (N=815)*
Safety Measures Scores*^a^			
0	20 (2%)	–	–
1	119 (10%)	–	–
2	441 (36%)	–	–
3	633 (52%)	–	–
Preparedness *S*cores**^b^			
0	4 (0.5%)	1 (0%)	0 (0%)
1	13 (1.5%)	6 (1%)	9 (1%)
2	45 (4%)	21 (3%)	14 (2%)
3	130 (13%)	72 (11%)	60 (10%)
4	326 (32%)	221 (33%)	180 (31%)
5	494 (49%)	352 (52%)	327 (56%)
Mean (SD)	4.22 (0.96)	4.32 (0.87)	4.36 (0.78)
Moderate to Severe Depression^c^	221 (18%)	111 (12%)	71 (9%)
Moderate to Severe Anxiety^d^	240 (20%)	125 (14%)	77 (10%)

^*^Safety Measures Score at wave 1: higher values indicate greater adherence to recommended safety measure behaviors.

^**^Preparedness Scores at concurrent wave: higher values indicate a higher level of preparedness.

a Missing values for safety measure scores: 9 (Wave 1).

b Missing values for preparedness scores: 210 (Wave 1), 254 (Wave 2), 225 (Wave 3).

c Missing values for moderate to severe depression: 6 (Wave 1), 5 (Wave 2), 10 (Wave 3).

d Missing values for moderate to severe anxiety: 1 (Wave 1), 5 (Wave 2), 6 (Wave 3).

### Association of participant characteristics with exposures and mental health outcomes

**Table 3** presents associations between demographic factors and exposures at Wave 1. A significant association was found between cohort types and safety measures scores (p=0.02). The SJ cohort had the highest mean safety measure scores (2.50, SD=0.71) across all three cohorts. Significant differences were observed for preparedness levels across demographic factors. Participants in the SJ cohort had significantly lower mean preparedness score (3.95, SD=1.12) compared to those in the SW (4.22, SD=0.88) and FR (4.29, SD=0.82) cohorts, indicating the lower preparedness. Preparedness levels varied significantly across age groups (p=0.02), with the 36-50 age group having the lowest mean preparedness score (3.99, SD=1.20) among all age groups. Hispanic participants had significantly the lower preparedness scores (3.99, SD=1.04) compared to other racial groups (p<0.001). Furthermore, low-income reported lower preparedness scores (3.79, SD=1.14) compared to those with high-income (4.35, SD=0.79). Preparedness levels also varied significantly across marital status groups (p=0.01), with divorced groups and those in other marital statuses showing lower preparedness scores (4.13, SD=0.91; 3.84, SD=1.21 respectively), compared to married/living with a partner (4.23, SD=0.91) and never married groups (4.22, SD=0.87).

**Table 3:** Association between demographic factors and exposures at wave 1.

Participant Characteristics/ Covariates	Safety measure Score^*^ Mean (SD)	P-value (t-test or ANOVA test)	Preparedness Score^**^ Mean (SD)	P-value (t-test or ANOVA test)
Type of Cohorts				
The First Respondents (FR)	2.35 (0.75)	**0.02**	4.29 (0.82)	**<0.001**
The Stress and Justice (SJ)	2.50 (0.71)		3.95 (1.12)	
The Stress and Wellbeing (SW)	2.36 (0.73)		4.22 (0.88)	
Type of participants				
Parents	2.40 (0.72)	0.72	4.19 (0.97)	0.91
Young adults	2.38 (0.75)		4.19 (0.85)	
Age				
15-25	2.33 (0.78)	0.15	4.19 (0.87)	**0.02**
26-35	2.45 (0.69)		4.14 (0.80)	
36-50	2.47 (0.73)		3.99(1.20)	
51-65	2.38 (0.72)		4.25 (0.87)	
66 and above	2.29 (0.71)		4.35 (0.88)	
Genders				
Male	2.33 (0.76)	0.06	4.24 (0.84)	0.45
Female	2.42 (0.72)		4.18 (0.96)	
Race and ethnicity^a^				
Asian	2.32 (0.67)	0.07	4.27 (0.94)	**<0.001**
Black or African American	2.53 (0.63)		4.14 (1.06)	
White, Not Hispanic	2.36 (0.72)		4.28 (0.81)	
Hispanic	2.44 (0.76)		3.99 (1.04)	
Other groups	2.28 (0.81)		4.33 (0.80)	
Household Income^b^				
Low	2.48 (0.70)	0.16	3.79 (1.14)	**<0.001**
Middle	2.38 (0.75)		4.15 (0.90)	
High	2.36 (0.73)		4.35 (0.79)	
Medical condition^c^				
Yes	2.41 (0.71)	0.52	4.16 (1.01)	0.36
No	2.38 (0.74)		4.23 (0.88)	
Marital status^d^				
Married, Living with a partner	2.41 (0.72)	0.55	4.23 (0.91)	**0.01**
Never married	2.37 (0.76)		4.22 (0.87)	
Divorced, Widowed, Separated	2.33 (0.71)		4.13 (0.91)	
Other marital statuses	2.32 (0.76)		3.84 (1.21)	

^*^Safety measures score: higher values indicate greater adherence to recommended safety measure behaviors. ^*^ ^*^ Preparedness score: higher values indicate a higher level of preparedness.

a Missing values for race and ethnicity: 15.

b Missing values for household income: 139.

c Missing values for medical condition: 175.

d Missing values for marital status: 1.

Bolded values indicate statistical significance at p<0.05.

As shown in **Table 4**, several demographic factors were associated with mental health outcomes. Household income was significantly associated with both depression (p=0.02) and anxiety (p=0.03), with higher proportions of those with depression (21%) and anxiety (22%) in low-income groups compared to those without depression (15%) and anxiety (14%). Gender was significantly associated with anxiety (p=0.04), with females comprising a larger proportion of those with anxiety (71%) compared to those without anxiety (61%). Medical condition was strongly associated with anxiety (p<0.001) and marginally associated with depression (p=0.08). Marital status was significantly associated with depression (p=0.01), with never married individuals composing a larger proportion of those with depression (43%) compared to those without depression (33%).

**Table 4:** Association between demographic factors and mental health outcomes at wave 1.

Characteristics/Covariates	DEPRESSION, Wave 1 N=1215			ANXIETY, Wave 1 N=1215		
	No - Depression (N= 994; 82%%) or Mean (SD)	Yes - Depression (N= 221; 18%) or Mean (SD)	P-values (Chi-Squares test or t-test)	No - Anxiety (N= 977; 80%) or Mean (SD)	Yes - Anxiety (N= 238; 20%) or Mean (SD)	P-values (Chi-Squares test or t-test)
Types of Cohorts						
FR	384 (39%)	78 (35%)	0.62	373 (38%)	89 (37%)	0.97
SJ	209 (21%)	47 (21%)		206 (21%)	50 (21%)	
SW	401 (40%)	96 (43%)		398 (41%)	99 (42%)	
Types of participants						
Parents	567 (57%)	116 (52%)	0.26	540 (55%)	142 (60%)	0.25
Young adults	428 (43%)	105 (48%)		437 (45%)	96 (40%)	
Age						
15-25	258 (26%)	63 (29%)	0.09	269 (27%)	52 (22%)	0.07
26-35	158 (16%)	42 (19%)		159 (16%)	41 (17%)	
36-50	133 (13%)	33 (15%)		124 (13%)	42 (18%)	
51-65	372 (38%)	77 (35%)		356 (37%)	93 (39%)	
66 and above	73 (7%)	6 (3%)		69(7%)	10 (4%)	
Genders						
Male	372 (37%)	77 (35%)	0.52	381 (39%)	68 (29%)	**0.04**
Female	622 (63%)	144 (65%)		596 (61%)	170 (71%)	
Race and ethnicity^a^						
Asian	63 (6%)	10 (5%)	0.06	64 (7%)	9 (3%)	**0.05**
Black or African American	68 (7%)	19 (9%)		68 (7%)	19 (8%)	
White, Not Hispanic	466 (47%)	86 (39%)		458 (47%)	94 (40%)	
Hispanic	279 (28%)	68 (30%)		269 (27%)	78 (33%)	
Other groups	118 (12%)	38 (17%)		118 (12%)	38 (16%)	
Household Income^b^						
Low	130 (15%)	42 (21%)	**0.02**	126 (14%)	46 (22%)	**0.03**
Middle	292 (33%)	74 (37%)		295 (34%)	71 (33%)	
High	469 (52%)	85 (42%)		457 (52%)	97 (45%)	
Medical condition^c^						
Yes	222 (26%)	64 (33%)	0.08	210 (25%)	76 (37%)	**<0.001**
No	625 (74%)	132 (67%)		628 (75%)	129 (63%)	
Marital status^d^						
Married, Living with a partner	487 (49%)	**87 (39%)**	**0.01**	460 (47%)	114 (48%)	0.86
Never married	328 (33%)	**94 (43%)**		344 (35%)	78 (33%)	
Divorced, Widowed, Separated	112 (11%)	**31 (14%)**		112 (12%)	31 (13%)	
Other marital status	66 (7%)	**9 (4%)**		60 (6%)	15 (6%)	

a Missing values for race: 15 for both depression and anxiety.

b Missing values for household income: 123 for both depression and anxiety.

c Missing values for medical condition: 172 for both depression and anxiety.

d Missing values for marital status: 1 for both depression and anxiety.

Bolded values indicate statistical significance at p<0.05.

### The impact of safety measures on individuals time trends of mental well-being

**Figure 1:**
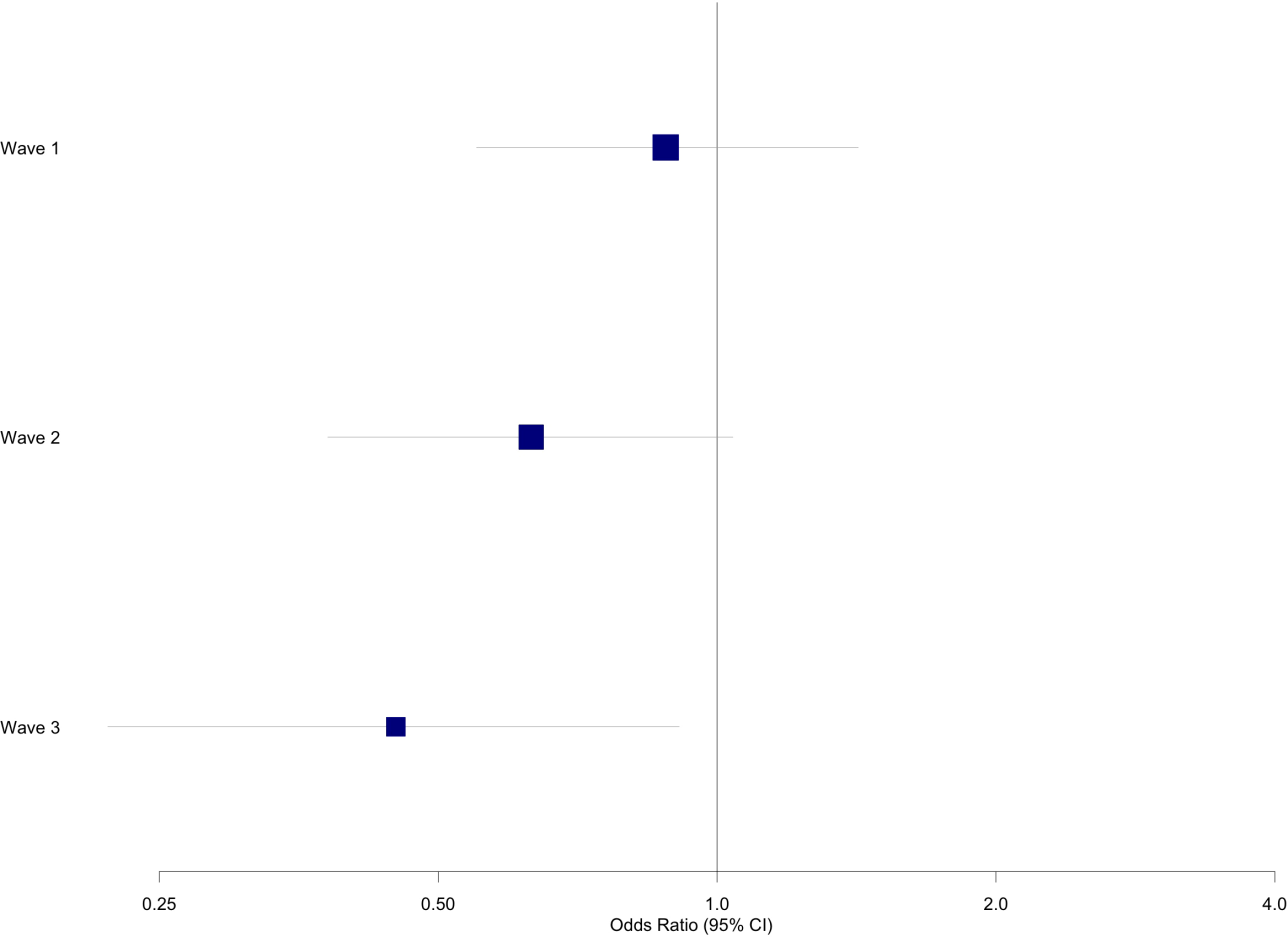
Odds ratios and confidence intervals for association of safety measures with depression across three waves.

As shown in **Table 5**, after covariate adjustment of gender and cohort types, the analysis revealed a significant interaction between safety measure scores and time for depression (aOR = 0.71, 95% CI: 0.51 to 0.99, p = 0.05). This indicates that the effect of safety measures on depression varied across the three waves. Specifically, the odds ratios for depression associated with Wave 1 safety measures decreased over time, with a reduction from Wave 1 (OR = 0.88, 95% CI: 0.55 to 1.42) to Wave 2 (OR = 0.63, 95% CI: 0.38 to 1.04), and a further reduction from Wave 2 to Wave 3 (OR = 0.45, 95% CI: 0.22 to 0.91) (**Figure 1**).

**Table 5:** Longitudinal analysis of the relationship between safety measures and mental health.

	DEPRESSION		ANXIETY	
	aOR (95% CI)	p-value	aOR (95% CI)	p-value
Safety Measure scores^*^	0.89 (0.56, 1.42)	0.61	1.01 (0.97, 1.05)	0.67
Wave	0.63 (0.29, 1.40)	0.26	**0.37** **(0.29, 0.47)**	**<0.001**
Safety Measure Scores x Wave	**0.71** **(0.51, 0.99)**	**0.05**	–	–
Male	1.00	Ref	1.00	Ref
Female	1.10 (0.54, 2.26)	0.79	1.70 (0.86, 3.36)	0.13
FR cohort	1.00	Ref	1.00	Ref
SJ cohort	1.34 (0.52, 3.41)	0.55	1.26 (0.53, 3.00)	0.60
SW cohort	1.23 (0.56, 2.67)	0.61	1.04 (0.50, 2.13)	0.93

aOR adjusted odds ratio: model adjusted for all other variables shown in the table.

^*^Safety measure score at wave 1: higher values indicate greater adherence to recommended safety measure behaviors.

Bolded values indicate statistical significance at p<0.05.

In contrast, the interaction term between safety measure scores and time was not significant for anxiety (p > 0.05). However, the main effect of time was significant (aOR = 0.37, 95% CI: 0.29, 0.47, p < 0.001), indicating a decrease in the likelihood of anxiety over time.

### Impact of preparedness levels on depression and anxiety

The relationship between preparedness and mental health outcomes was examined adjusting for income and marital status (**Table 6**). The analysis revealed a significant negative effect of time on both anxiety and depression (aOR=0.27, 95% CI: 0.20, 0.36; p<0.001 and aOR=0.37, 95% CI: 0.29, 0.48; p<0.001, respectively), indicating a decrease in depression and anxiety levels over the three waves. The interaction terms between wave and preparedness were initially tested but found to be non-significant (p>0.05) for both depression and anxiety models.

**Table 6:** Longitudinal analysis of the relationship between preparedness and mental health.

	DEPRESSION		ANXIETY	
	aOR (95% CI)	p-value	aOR (95% CI)	p-value
Preparedness scores^*^	1.08 (0.72, 1.45)	0.65	**0.72** **(0.55, 0.93)**	**0.01**
Wave	**0.27** **(0.20, 0.36)**	**< 0.001**	**0.37** **(0.29, 0.48)**	**<0.001**
Low Household Income	1.00	Ref.	1.00	Ref.
Middle Household Income	0.54 (0.18, 1.61)	0.27	0.44 (0.15, 1.26)	0.13
High Household Income	0.40 (0.13, 1.18)	0.10	**0.35** **(0.12, 1.00)**	**0.05**
Divorced; Widowed; Separated	1.00	Ref.	1.00	Ref.
Married and living with *sp*ouse	0.71 (0.22, 2.34)	0.58	0.88 (0.29, 2.62)	0.81
Never married	1.12 (0.34, 3.68)	0.85	0.75 (0.25, 2.31)	0.62
Other marital status	0.72 (0.11, 4.86)	0.74	0.67 (0.11, 4.00)	0.66

aOR adjusted odds ratio: model adjusted for all other variables shown in the table.

^*^ Preparedness score at concurrent wave: higher values indicate a higher level of preparedness.

Bolded values indicate statistical significance at p<0.05.

The preparedness score showed differential associations with mental health outcomes. For depression, no significant association was observed. However, preparedness score significantly associated with anxiety (aOR=0.72, 95% CI: 0.55, 0.93; p=0.01), with higher preparedness scores (indicating higher level of preparedness) corresponding to reduced odds of anxiety. Socioeconomic status was independently associated with anxiety outcomes, with participants from high income household showing marginally significant lower odds of anxiety (aOR=0.35, 95% CI: 0.12, 1.00; p=0.05) compared to low income participants.

### Variations in mental health outcomes associations with safety measures and preparedness across age, race/ethnicity and income

The relationship between safety measures and mental health outcomes was examined across participant characteristics. The base model, which included demographic factors race, age, and income, demonstrated a significant association between high household income and lower odds of anxiety (OR=0.26, 95% CI: 0.08, 0.87; p=0.03). However, interaction models assessing the relationship between safety measures and mental health outcomes separately for each demographic factor did not reveal any significant interactions (all p>0.05).

**Figure 2:**
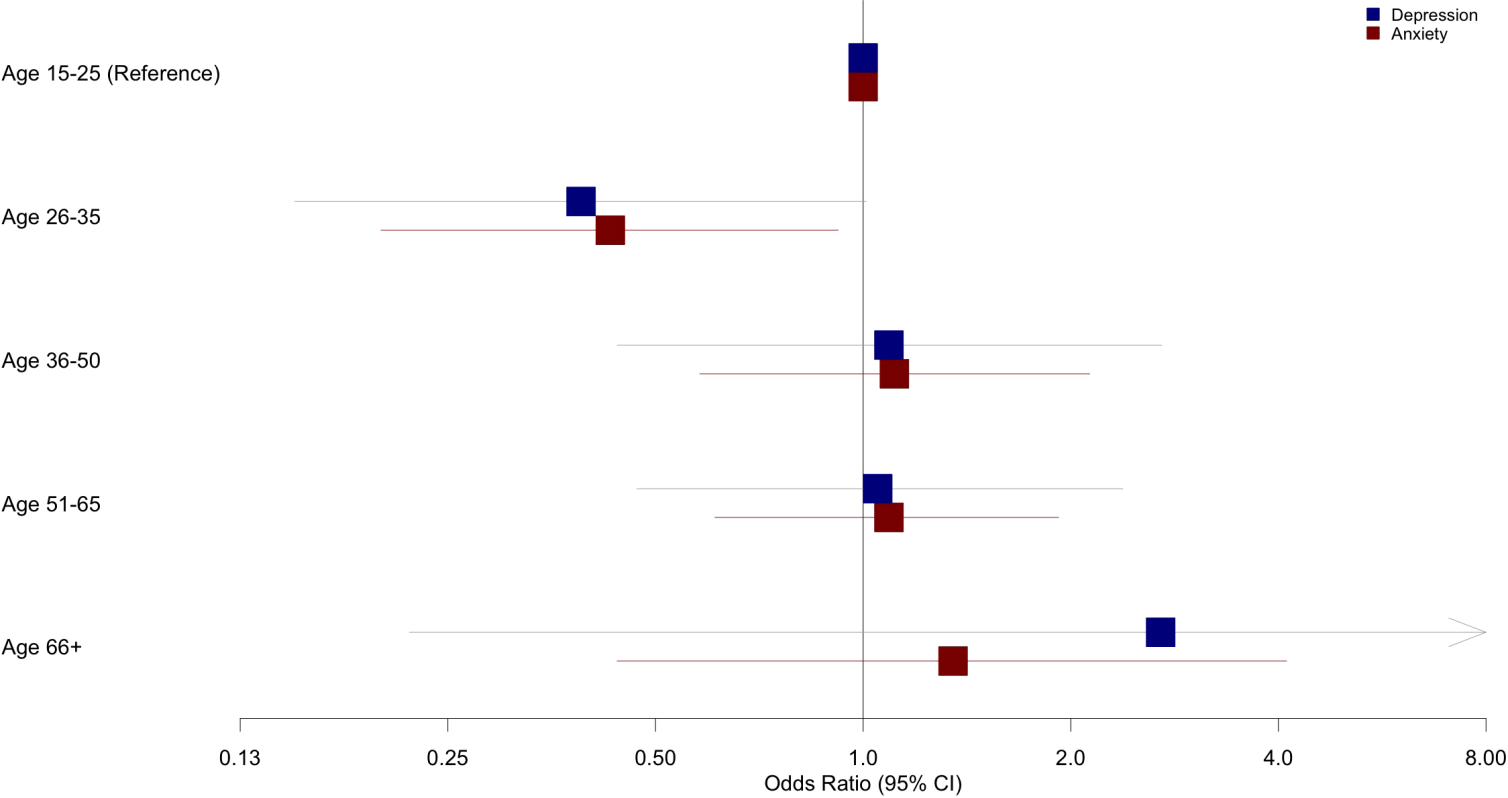
Age-specific association of preparedness with depression and anxiety.

In contrast, the relationship between preparedness and mental health outcomes showed significant variation across age groups. Specifically, the age interaction model (**Table 7**) revealed a significant interaction between preparedness scores and the 26-35 age group for anxiety (OR=0.43, 95% CI: 0.2, 0.92; p=0.03), and a marginally significant interaction for depression (OR=0.15, 95% CI: 0.15, 1.01; p=0.05). This indicates that among participants aged 26-35, higher levels of preparedness were significantly reduced odds of anxiety and marginally reduced odds of depression compared to the reference group aged 15-25. No other age groups demonstrated significant preparedness interactions, indicating the protective effect of preparedness was specific to the 26-35 age group (**Figure 2**).

**Table 7:** Preparedness and mental health outcomes: age-specific interaction models.

Terms	DEPRESSION		ANXIETY	
	OR (95% CI)	p-value	OR (95% CI)	p-value
Preparedness Scores^**^	1.20 (0.64, 2.27)	0.56	0.80 (0.51, 1.25)	0.34
**Preparedness x Age 26 - 35**	**0.39** **(0.15, 1.01)**	**0.05**	**0.43** **(0.2, 0.92)**	**0.03**
Preparedness x Age 36 - 50	1.09 (0.44, 2.71)	0.86	1.11 (0.58, 2.13)	0.76
Preparedness x Age 51 - 65	1.05 (0.47, 2.38)	0.90	1.09 (0.61, 1.92)	0.78
Preparedness **x** Age 66 and above	2.70 (0.22, 33.33)	0.44	1.35 (0.44, 4.17)	0.61
Age 15 - 25	1.00	Ref.	1.00	Ref.
Age 26 - 35	0.56 (0.13, 2.51)	0.45	1.30 (0.38, 4.48)	0.68
Age 36 - 50	0.99 (0.17, 5.66)	0.99	2.61 (0.59, 11.50)	0.21
Age 51 - 65	1.16 (0.23, 5.97)	0.86	2.69 (0.63, 11.43)	0.18
Age 66 and above	0.71 (0.07, 7.51)	0.78	1.41 (0.21, 9.49)	0.72
Wave	0.26 (0.20, 0.36)	<0.001	0.45 (0.36, 0.55)	<0.001
Asian	1.00	Ref.	1.00	Ref.
Black or African	1.39 (0.17, 11.30)	0.76	1.16 (0.17, 8.21)	0.88
Hispanic	1.21 (0.22, 6.75)	0.83	1.13 (0.23, 5.46)	0.88
Other races	1.93 (0.31, 12.06)	0.48	2.06 (0.38, 11.26)	0.41
White	1.12 (0.21, 5.96)	0.89	1.01 (0.22, 4.63)	1.00
Low income	1.00	Ref.	1.00	Ref.
Middle income	0.51 (0.16, 1.58)	0.24	0.42 (0.14, 1.25)	0.12
High income	0.38 (0.11, 1.28)	0.12	0.35 (0.11, 1.11)	0.07
Divorced, Widowed, Separated	1.00	Ref.	1.00	Ref.
Married and living with *sp*ouse	0.64 (0.19, 2.19)	0.48	0.87 (0.29, 2.62)	0.80
Never married	0.96 (0.19, 4.95)	0.96	1.27 (0.27, 5.90)	0.76
Other marital status	0.66 (0.08, 5.25)	0.70	0.96 (0.14, 6.70)	0.97

^**^Preparedness score at concurrent wave: higher values indicate a higher level of preparedness.

Bolded values indicate statistical significance at p<0.05.

## Discussion

Building on the broader pattern observed globally, this longitudinal study revealed complex relationships between safety measures, preparedness and mental health outcomes during the COVID-19 pandemic in NYC. Safety measures demonstrated a time dependent protective impact on depression. At Wave 1, while no significant association was observed, the protective effect of safety measures strengthened by Wave 2, and becoming statistically significant by Wave 3. This pattern, visualized in **Figure 1**, suggests a potential gradual and cumulative benefit of safety measures on reducing depressive symptoms over time. Multiple factors may have influenced this pattern. The adherence to safety measures might be associated with the development of adaptive coping strategies, potentially relating to feeling of control and self-efficacy (33), although other unmeasured factors could also play important roles. Additionally, the accumulation of knowledge about the virus and the effectiveness of preventive measures may have contributed to a reduction in perceived threat, thereby alleviating depressive symptoms (34, 35). In contrast, the effect of baseline safety measures on anxiety remained stable across all three waves, with no significant change observed. This finding aligns with studies from Germany and the United Kingdom, which reported consistent anxiety levels over time (36, 37).

Preparedness emerged as a critical factor influencing anxiety throughout the study period. Individuals who reported higher levels of preparedness consistently demonstrated lower odds of anxiety across all waves, underscoring the importance of readiness and access to essential resources in mitigating psychological distress during the COVID-19 pandemic. These findings aligned with existing literature indicating that perceived preparedness can enhance feeling of control and resilience, thereby reducing anxiety in crisis situation (38). Socioeconomic status independently influenced anxiety, with participants from high income household showing significant lower anxiety levels compared to their low-income counterparts. This finding aligns with the observed difference in preparedness levels, where low household income reported lower preparedness scores (**Table 2**). This highlighted how economic inequities compounded psychological vulnerabilities during the pandemic (23, 39).While no significant interaction effects were observed between race/ethnicity and mental health outcomes in longitudinal analyses, bivariate tests revealed racial disparity in preparedness on anxiety at baseline, with Hispanic participants had significantly lower preparedness scores compared to other racial groups (**Table 3**). This disparity reflects broader systemic inequities, such as reduced access to resources or heightened economic stressors, which could exacerbate psychological vulnerabilities during emergencies (25). Although the protective effects of preparedness on mental health outcomes appeared consistent across racial/ethnic groups in this study, these findings underscore the importance of addressing resource disparities to promote equitable mental health outcomes during public health emergencies (23, 40).

Additionally, the impact of preparedness on mental health outcomes varied significantly across different age groups, reflecting that age related developmental difference may influence psychological responses during crises (41). This demographic often experiences significant developmental transitions such as career establishment, financial independence, and family formation (42, 43), introducing heightened responsibilities and stressors, potentially amplifying vulnerability to psychological distress during public health emergencies (42). Previous research supported this perspective, indicating that working age adults (including ages 26–35) often exhibit greater susceptibility to psychological distress during pandemics compared to older populations (27, 41, 44), likely due to these unique pressures associated with early adulthood transitions (42). Preparedness emerged as a resilience enhancing factor for this group (43), likely due to its role in addressing practical concerns and reducing uncertainty (45). Within this age demographic, higher preparedness was associated with significantly lower anxiety and marginally lower depression. In contrast, no other age groups showed significant variations in the relationship between preparedness and anxiety, suggesting that the psychological benefits may be more pronounced in specific life phases (42). Further research is needed to understand the specific factors influencing this outcome. The current study provides additional insight by identifying preparedness as a critical psychological resource specifically for the younger segment. Recognizing the differential impact of preparedness across age groups underscores the importance of tailoring interventions to address the unique psychological and practical stressors experienced by young adults during prolonged public health crises.

The observed associations discussed above occurred in the context of declining in moderate and severe depression and anxiety over three waves in this study, aligning with global research highlighting the heterogeneous mental health trajectories observed throughout the pandemic (46, 47). While some populations experienced sustained distress, other demonstrated signs of psychological adaption and recovery (48). These variations were likely influenced by differences in coping mechanisms, public health responses, and lock down measures (48) was well as underlying demographic and socioeconomic factors that shaped mental health trajectories (49). These findings underscore the importance of tailoring interventions to address both individuals level risk factors and broader structural inequities.

### Strengths and limitations

This study benefits from a longitudinal design, featuring a diverse participant pool and standardized measures for assessing depression and anxiety. The three-wave data collection approach enabled the examination of temporal trends in mental health outcomes, providing valuable insights into the dynamic relationships between safety measures, preparedness, and mental health during the COVID-19 pandemic.

However, several limitations warrant consideration. First, the study experienced a decline in participation over the three waves, this may have introduced selection bias if those who dropped out differed systematically from those who remained in terms of baseline characteristics or time trends in mental well-being outcomes. Similarly, results could have been biased if participants with missing data had such systematic differences relative to those with complete data. Second, while the participant pool was diverse, it may not fully reflect the demographic composition of NYC’s population. In particular, certain minority groups, such as Asian participants were underrepresented in this sample, which may have limited statistical power for subgroups analyses. Additionally, while the study offers insights relevant to broader global concerns, its focus on an urban, multicultural US population may constrain its applicability to other settings. These factors may limit the applicability of the results to low- and middle-income countries or other settings with more homogeneous populations, where social, economic, and cultural contexts may differ significantly. Despite these limitations, this study contributes meaningfully to the understanding of the effects of specific COVID-19-related safety measures and preparedness on mental health outcomes during the pandemic, offering evidence for public health strategies.

## Conclusion

Consistent adherence to safety measures could mitigate depressive symptom over time while higher preparedness levels were significantly associated with reduced anxiety, particularly among adults aged 26-35. High income independently predicted lower anxiety, highlighting the potential role of both preparedness interventions, and financial stability mental health outcomes during prolonged crisis. These findings emphasize the importance of integrating both practical and psychological aspect of preparedness and safety measures into public health strategies.

## Data Availability

The data analyzed in this study is subject to the following licenses/restrictions: The dataset used in this study is not publicly available but is obtainable upon reasonable request and subject to appropriate data sharing agreements. Requests to access these datasets should be directed to Christina W. Hoven, ch42@columbia.edu.
